# Feasibility Testing of the Automatic Design of Three-Unit Implant Fixed Dental Prostheses with Different Dental CAD Software: A Pre-Clinical Pilot Trial

**DOI:** 10.3390/jcm14010233

**Published:** 2025-01-03

**Authors:** Selina A. Bernauer, Philipp Wieland, Nicola U. Zitzmann, Tim Joda

**Affiliations:** 1 Department of Reconstructive Dentistry, UZB University Center for Dental Medicine Basel, University of Basel, 4058 Basel, Switzerland; selina.bernauer@unibas.ch (S.A.B.); philipp.wieland@stud.unibas.ch (P.W.); n.zitzmann@unibas.ch (N.U.Z.); 2 Clinic of Reconstructive Dentistry, Center for Dental Medicine, University of Zurich, 8032 Zurich, Switzerland

**Keywords:** fixed prosthodontics, full crown, intraoral optical scanning (IOS), digital dentistry, CAD-CAM

## Abstract

**Background/Objectives:** The technical development of implant-supported fixed dental prostheses (iFDP) initially concentrated on the computer-aided manufacturing of prosthetic restorations (CAM). Advances in information technologies have shifted the focus for optimizing digital workflows to AI-based processes for design (CAD). This pre-clinical pilot trial investigated the feasibility of the automatic design of three-unit iFDPs using CAD software (Dental Manger 2021, 3Shape; DentalCAD 3.1 Rijeka, exocad GmbH). **Methods:** Two clinical scenarios based on a full dentition were created virtually. Physical models were produced and digitized using two intraoral scanners applying quadrant or full-arch scans (Trios3, 3Shape, Copenhagen, Denmark; and Primescan AC, Dentsply Sirona, Bensheim, Germany). For each scenario, iFDP designs were generated automatically using two laboratory software systems (Dental Manger 2021, 3Shape; DentalCAD 3.1 Rijeka, exocad GmbH), resulting in 80 STL datasets (2 scenarios × 2 scan strategies × 2 IOS systems × 5 scan repetitions × 2 software). The files were analyzed clinically for the contact schemes and pontic area. One of the automated designs for each scenario was manually post-processed and one iFDP design for each scenario was manually created by experienced dental technicians (control). The time required for all the design processes was recorded. **Results:** The automatic design of iFDPs without manual adjustment did not lead to clinically acceptable restorations. The time required for the automatically generated/manually adjusted iFDPs designs was not significantly different to that for the manually designed restorations. **Conclusions:** Current laboratory software can not automatically generate three-unit iFDPs with clinically acceptable results in terms of the interproximal and occlusal contacts and the pontic design. The automatic iFDP design process currently requires manual adjustment, which means there is no benefit in terms of the working time compared with manually created restorations.

## 1. Introduction

Digitization has become an integral part of society. The so-called Generation Z, individuals born between 1997 and 2012, is currently shaping sociopolitical developments. They are the first generation to grow up with smartphones and described as digital natives. They use social media and artificial intelligence (AI) online tools as a matter of course [1]. It is not surprising that the use of digital technology is not only part of everyday life but has also become an indispensable part of dentistry. Especially in fixed prosthetics, it has paved the way for simplified clinical and laboratory workflows with new technological possibilities [2]. The adoption of intraoral scanning (IOS) and computer-aided design/computer-aided manufacturing (CAD-CAM) has significantly changed dental protocols in recent years [3].

Digital workflows can be divided into three main work steps: (i) digitalization of the individual patient situation using IOS; (ii) design of the prosthetic restoration with dental laboratory software (CAD); and (iii) manufacture of the prosthesis by rapid prototyping (CAM). High data volumes must be transferred during this workflow, and different technologies are used to forward datasets from IOS software to CAD/CAM applications. If the data transfer only remains within the hardware and software of one manufacturer, this workflow is referred to as proprietary. A special case is when the end user has no access to the generated data, which is referred to as a closed system. It is used primarily to manufacture small units of fixed dental prostheses, such as inlays, onlays, and crowns. The software automatically designs the desired restoration, either as a biogeneric copy or as a biogeneric individual [4,5]. One illustrative example is the CEREC system (Dentsply Sirona, Bensheim, Germany), which stands for CEramic REConstruction. These types of restorations have already shown promising results in terms of accuracy and efficiency compared to conventional methods [6]. Multi-unit restorations often require external software for the CAD process. To transfer data between IOS and external software, an open system using the Standard Tessellation Language (.STL) is required, as it can be read by all different programs. It has been shown that no data is lost during transfer when output programs are integrated [7].

A complete digital workflow is defined as a clinical and technical process without the production of any physical dental models. Complete digital workflows have demonstrated high-quality outcomes for single-tooth restorations and fixed dental prostheses on teeth and implants in terms of accuracy, patient satisfaction, and economics, as well as their time-efficiency and production costs [8,9,10]. Literature reviews have shown the superiority of complete digital workflows over other technologies; however, digital dental design is still a manual process [9,11]. The question is whether an AI-based restorative design process offers economic and perhaps even qualitative advantages.

Therefore, the aim of this pre-clinical pilot trial was to investigate whether current CAD-CAM software is able to automatically generate clinically acceptable designs of implant-supported fixed dental prostheses (iFDP), based on the information available in the form of full-arch or quadrant scans, using two clinical scenarios, i.e., a tooth-supported gap and a free-end situation in posterior sites. A specific focus was placed on the occlusal contacts, the interproximal contact schemes, and the pontic design. Secondly, the time required by experienced dental technicians to (i) manually improve the automatically generated designs, and (ii) fabricate the designs for the three-unit iFDPs completely manually, was evaluated.

The first hypothesis was that CAD-CAM lab software can automatically create clinically acceptable iFDP designs regarding interproximal contact schemes, the pontic design, and occlusal morphologies, considering different baseline situations. The second hypothesis was that there is no difference between the automatically or manually designed three-unit iFDPs in terms of the CAD-time.

## 2. Material & Methods

### 2.1. Study Setup

A fully dentate patient, whose case has been anonymized and further modified to create an ideal model situation, was intraorally digitized using IOS (Trios 3, 3Shape, Copenhagen, Denmark). The dental and oral conditions were considered as healthy with stable interocclusal relationships, no prosthetic or orthodontic appliances, and minimal or no occlusal wear. Teeth 14, 15, and 16 were virtually erased and two implants placed in the first molar region (area 16, Straumann TL WN 4.8 mm, Institute Straumann AG, Basel, Switzerland) and the first premolar region (area 14, Straumann TL RN 4.1 mm). Implants were positioned following the principles of prosthetically driven backward planning for a prospective iFDP. Based on this modified clinical situation, physical models were 3D printed (3D Printer, Sheraflash-light Material). Models were arbitrarily mounted in an analog articulator (SAM3 Articulator, SAM Praezisionstechnik GmbH, Gauting Germany) and served as reference models. The terminal second molar (tooth 17) was removable, enabling two different situations as a free-end situation or a toothed area.

### 2.2. Study Setting

The application of different scanning strategies with full-arch or quadrant scanning to the two situations resulted in four groups:

- A. Full-arch scan of the upper and lower jaws including bilateral bite registration with tooth 17;
- B. Full-arch scan of the upper and lower jaws including bilateral bite registration without tooth 17;
- C. Quadrant scan of the upper and lower jaws including unilateral bite registration with tooth 17;
- D. Quadrant scan of the upper and lower jaws including unilateral bite registration without tooth 17.

**Figure 1:**
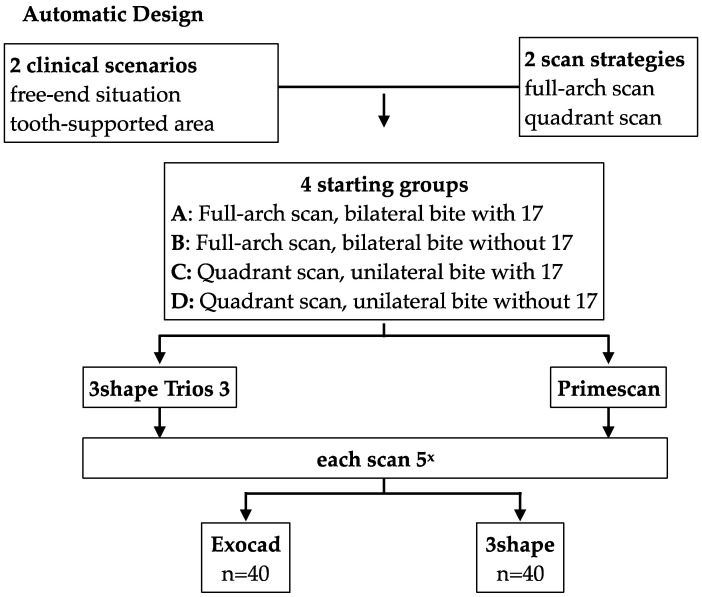
Study design.

Scanning of these four groups was repeated five times each with two different IOS systems (Trios 3, 3Shape, Copenhagen, Denmark and CEREC Primescan AC, Dentsply Sirona, Bensheim, Germany), resulting in a total of 40 different scans. Subsequently, these scans were saved as STL files. The obtained STL datasets were then further processed in two types of CAD software (Dental Manger 2021, 3Shape; DentalCAD 3.1 Rijeka, exocad GmbH). For each of these scans a three-unit iFDP was automatically designed for implants 14 and 16 and then the designs were saved as new STL files. This resulted in a total of 80 designs/STL files (2 scenarios × 2 scan strategies × 5 scan repetitions × 2 IOS systems × 2 types laboratory software). The study setting is visualized in Figure 1. All the practical work steps were performed by the same operator (P.W.), a postgraduate dentist. Each step was supervised by a senior clinician (S.A.B.).

### 2.3. Manual Adjustments, Time Evaluation, and Visual Examination

All these 80 automatically designed iFDPs were visually examined for their clinically applicable design, focusing on the interproximal contacts, occlusal contacts, and pontic design. To achieve such a design, the iFDP should exhibit regularly distributed occlusal contacts in maximum intercuspation and group function during dynamic movements. Additionally, it must ensure proximal contact with neighboring teeth and feature an ovate pontic design. Since the results already visually deviated significantly from the aforementioned points, no further comparisons with superimposition or a matching program were made.

Further, one randomly selected scan, as an STL file out of each group (A-D), was used to manually generate an iFDP by an experienced dental technician (R.S.B. or J.C.). Another randomly selected scan, as an STL file, was used for the automatic iFDP design and manual adjustments. The time required by the technician to (a) produce the clinically ideal iFDP design only manually, (b) generate the iFDP design automatically by the software, and (c) improve the iFDP design automatically generated by the software so that they could be used clinically, was evaluated. The use of quadrant and full scans in two clinical scenarios, applying two laboratory processes, equaled a total of 16 manually generated or manually adjusted iFDP designs, 8 designs per dental technician.

### 2.4. Statistical Analyses

The collected time data for the 16 manually designed or adjusted iFDPs were analyzed descriptively by calculating the mean times and standard deviations (±SD), as well as the minimum and maximum values. These data were calculated individually for the complete manual design, the complete automatic design, and the automatic plus improved design, in the form of a two-sample *T*-test for both the programs, Exocad and 3Shape. The significant difference in the time required by the two programs was then analyzed using a paired two-sample *T*-test for the means.

## 3. Results

### 3.1. Results of the Automatic CAD Process

**Figure 2:**
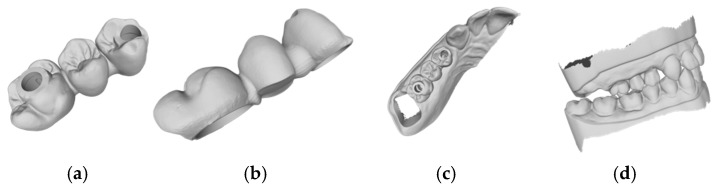
Example of an automatic design using Exocad lab software (DentalCAD 3.1 Rijeka, exocad GmbH): (**a**) occlusal examination of the bridge design; (**b**) vestibular evaluation of the bridge design; (**c**) occlusal evaluation on the dental arch; (**d**) occlusal relations with the antagonist arch.

**Figure 3:**
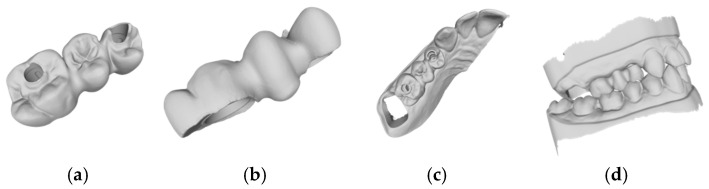
Example of an automatic design using 3Shape lab software (Dental Manger 2021, 3Shape): (**a**) occlusal examination of the bridge design; (**b**) vestibular evaluation of the bridge design; (**c**) occlusal evaluation on the dental arch; (**d**) occlusal relations with the antagonist arch.

Based on the criteria for the objective assessment (interproximal contacts, occlusal contacts, and pontic design), none of the completely automatic designs of the three-unit iFDPs were clinically acceptable, which was already evident by visual inspection. This was due to an inadequate crown emergence profile, an insufficient connector size (Figure 2b and Figure 3b), the unacceptable position of the screw access, interference with the occlusal relief resulting in excessive occlusion (Figure 2a and Figure 3a), the absence of approximal contacts, and a bridge design not in line with the dental arch circumference (Figure 2c,d and Figure 3c,d). Due to the lack of accuracy and the clinically unacceptable designs, no further comparisons were made with these designs.

### 3.2. Time Efficiency

**Figure 4:**
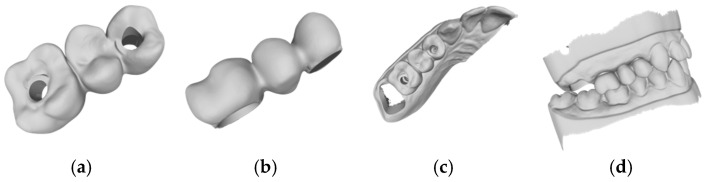
Example of an automatic design with manual adjustment using Exocad lab software: (**a**) occlusal examination of the bridge design; (**b**) vestibular evaluation of the bridge design; (**c**) occlusal evaluation on the dental arch; (**d**) occlusal relations with the antagonist arch.

**Figure 5:**
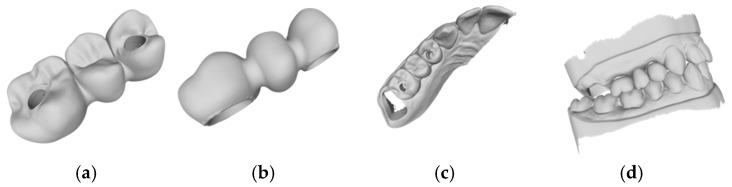
Example of a manual design using Exocad lab software: (**a**) occlusal examination of the bridge design; (**b**) vestibular evaluation of the bridge design; (**c**) occlusal evaluation on the dental arch; (**d**) occlusal relations with the antagonist arch.

**Figure 6:**
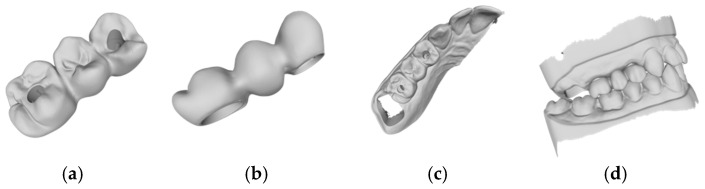
Example of an automatic design with manual adjustment using 3Shape lab software: (**a**) occlusal examination of the bridge design; (**b**) vestibular evaluation of the bridge design; (**c**) occlusal evaluation on the dental arch; (**d**) occlusal relations with the antagonist arch.

**Figure 7:**
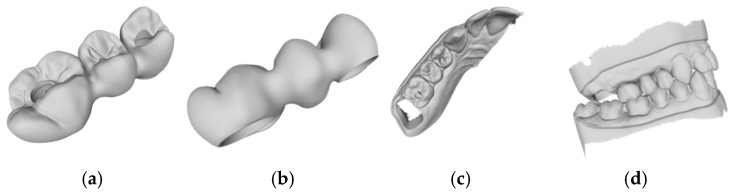
Example of a manual design using 3Shape lab software: (**a**) occlusal examination of the bridge design; (**b**) vestibular evaluation of the bridge design; (**c**) occlusal evaluation on the dental arch; (**d**) occlusal relations with the antagonist arch.

**Figure 8:**
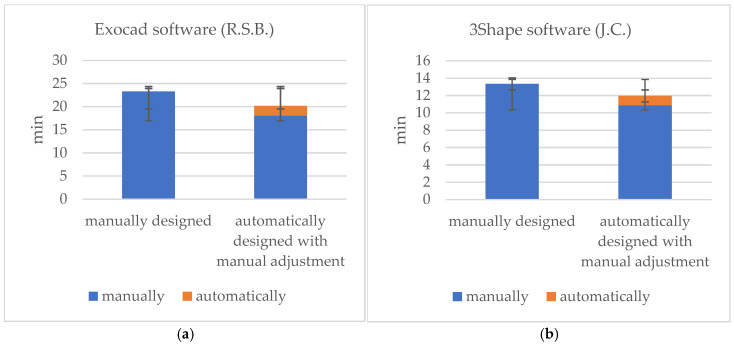
Visual representation illustrating the dental technician’s time efficiency in creating the design manually versus adjusting the automatically generated design. (**a**) Mean time using the Exocad software, (**b**) mean time using the 3Shape software.

The times recorded for the completely manual designs and the manual adjustments of the automatic designs are shown in Table 1. For the Exocad software (DentalCAD 3.1 Rijeka, exocad GmbH), the mean time requirements were 23.3 (±2.3) min for the manual design, 2.1 (±0.7) min for the automatic design, and 18.0 (±3.1) min for the automatic design with manual adjustment (Figure 2, Figure 4 and Figure 5). The mean times for the 3Shape software (Dental Manger 2021, 3Shape) were 13.4 (±3.3) min for the manual design, 1.1 (±0.1) min for the automatic design, and 10.9 (±1.5) min for the manual adjustment (Figure 3, Figure 6 and Figure 7). Since the automatic designs did not produce clinically acceptable results, the time of the manual adjustment was added to the time of the automatic design, with results of 12.0 (±1.4) min for the 3Shape software and 20.2 (±2.5) min for the Exocad software (Figure 8).

Time-comparisons for the completely manually produced restorations and those automatically generated with manual adjustments revealed no statistically significant differences (*p* = 0.18).

## 4. Discussion

### 4.1. Objective Assessment

The current in vitro study compared CAD-CAM-based automatic and manual designs of iFDPs and demonstrated that no clinically acceptable designs of the automatically generated iFDP projects were discerned, even through visual inspection. In consideration of these findings, the null hypothesis that no difference existed between the automatic and manual design of three-unit implant-supported iFDPs was rejected. A comparison of the automatically generated designs was not reasonable due to the visually evident deviations from the objective assessment criteria, such as occlusal contacts, interproximal contacts, and the pontic design.

Kim et al. highlighted that, in the context of occlusal contacts, osseointegrated implants respond biomechanically differently to occlusal forces compared to natural teeth, because of the absence of the periodontal ligament. It is therefore believed that dental implants are more susceptible to occlusal overloading, a factor recognized as a major contributor to peri-implant bone loss, ultimately leading to the failure of implants and implant-supported restorations [12]. According to Rachel et al., the recommendation for occlusal schemes in iFDPs in the posterior region includes a mutually protected occlusion with anterior guidance and evenly distributed contacts, allowing for wide freedom in centric relation [13]. Another common multifactorial implant complication, as described by Saber et al., is interproximal contact loss, which has been identified as correlating with marginal bone loss [14,15]. Furthermore, the design of the pontic is a crucial component of a clinically acceptable iFDP. In the posterior segment, where esthetics are less critical, an ovate pontic form is most compatible with function and hygiene [16]. Unfortunately, Kazmi et al. concluded that a significant portion of dentists do not fully adhere to the guidelines for a sufficient pontic design [17]. Due to the high risk of complications associated with these objective assessment criteria, the automatically generated iFDPs in this study are deemed clinically unacceptable. Consequently, the expertise of a dental technician remains indispensable in current practice.

### 4.2. Automatic Design

Considering single specific parameters and/or other software for the automatic CAD design, Arslan et al. noted, for example, that a natural occlusal relief can be achieved with automatic processes in CEREC software using the biogeneric design modes [18]. These findings are corroborated by the results of a study conducted by Litzenburger et al. [19]. The opposing outcomes relative to this study may be attributed to the proprietary workflow employed in the CEREC system. Furthermore, these studies utilized natural teeth rather than implants. It would be interesting to conduct a study using iFDPs instead of natural teeth to investigate potential differences compared to the present study.

Digital crown design entails the extraction of biological characteristic curves, the retrieval of specific elements from a tooth library, and the precise adjustment of the morphology [20]. The algorithms employed by different CAD software systems exhibit notable variations. For instance, 3Shape and CEREC extract feature lines by identifying a sequence of biological characteristic curves, while Exocad necessitates the addition of multiple interaction points to refine the initially extracted feature line [21]. By considering different scanning strategies and clinical scenarios, this study aimed to determine whether additional information in the scan or scenario would result in improved design outcomes. Yu et al., in a previous study, demonstrated a higher accuracy of virtual interocclusal records with quadrant scans compared to full-arch scans [22]. Simultaneously, Wang et al. demonstrated that missing adjacent teeth and abrasion resulted in a greater loss of characteristic features [21]. However, in our study, no differences were observed between the various scanning strategies and scenarios, as none of the designs achieved clinically acceptable outcomes.

### 4.3. Time Efficiency

A fully digital workflow was previously applied, but manual adjustment to the CAD design was required to achieve clinically acceptable results. In terms of time efficiency, our findings are supported by Bernauer et al., in that a fully digital workflow can at least be comparable to a conventional or a hybrid workflow [9]. However, as manual adjustment of the automatic designs is still necessary, no time savings are currently possible. Further development of the laboratory software is necessary to produce fully automatically designed iFDPs, a process which is currently in demand within the industry.

In the current study, the time required to produce an iFDP was not significantly affected by whether it was designed manually or improved from an automatic design. However, it is important to note that once a design has been automatically created, there were fewer restructuring tools available than when each individual step is designed manually. This applied equally to both laboratory programs. Consequently, it was more straightforward to construct a new iFDP without any pre-existing design.

### 4.4. Artificial Intelligence

Recent studies have shown promising results using deep learning programs, a subset of artificial intelligence (AI), in dental design. As reported by Cho et al., implant-supported crowns (ISCs) designed using deep learning programs demonstrated an occlusal table area, cusp angle, cusp height, proximal contact, and emergence profile angle comparable to those designed by a dental technician, achieving clinically acceptable results. Additionally, significant differences were observed in the time required for the ISC design between the automated AI-based approach and the manual design by a dental technician [23,24]. Chau et al. further demonstrated that a 3D GAN AI system could accurately replicate biometric crown designs [25]. However, further developments are necessary to successfully integrate these AI algorithms into the software used in this study.

At present, there are web-based cloud solutions that offer promising CAD for single-crown restorations “www.dentbird.com (accessed on 1 December 2024)”. Such developments may prove significant in the context of ongoing digital globalization.

### 4.5. Limitations

The small number of CAD restorations designed by the dental technicians was a possible limitation of this pre-clinical pilot project. A larger dataset would allow for a more comprehensive analysis, which could be conducted in a future study. For both types of the CAD software used, updated versions are now available. According to Exocad, the new version, Dental CAD 3.2 Elefsina, should include more automation and time-saving features, which could impact the outcome of the results from the current study. General improvements to Dental Manager and Dental Designer are also described for the new 3Shape Dental System 2023 version.

### 4.6. Future

The potential for future clinical research to be conducted based on newer, more advanced, or different software, which offers enhanced capabilities, should be acknowledged. A reliable AI algorithm for the CAD of iFDPs could offer advantages even with chairside, one-visit solutions. Overall, these restorations would most likely be more cost and time effective. It seems feasible that CAD software will become capable of producing complete restorations without any manual input in the future.

## 5. Conclusions

Within the limitations of this study, the following conclusions can be drawn:

1. The tested software systems were unable to automatically generate iFDP designs with clinically acceptable parameters.
2. The manual adjustment of the automatically generated iFDP designs did not result in any significant difference in time efficiency compared to a fully manual design.

## Figures and Tables

**Table 1:** Time required for the design in groups A–D.

Exocad (R.S.B.)	Manually	Automatically	Adjustment Time	3Shape (J.C.)	Manually	Automatically	Adjustment Time
Group A	22 min 51 s	2 min 59 s	13 min 21 s	Group A	18 min 58 s	1 min 10 s	9 min 44 s
Group B	19 min 56 s	1 min 37 s	19 min 47 s	Group B	12 min 32 s	1 min 12 s	9 min 09 s
Group C	23 min 58 s	1 min 20 s	21 min 41 s	Group C	10 min 37 s	1 min 05 s	12 min 28 s
Group D	26 min 23 s	2 min 33 s	17 min 21 s	Group D	11 min 16 s	54 s	12 min 08 s

## Data Availability

All relevant data are presented in this manuscript. No additional data source is available.
